# SERIES: eHealth in primary care. Part 2: Exploring the ethical implications of its application in primary care practice

**DOI:** 10.1080/13814788.2019.1678958

**Published:** 2019-10-30

**Authors:** Sarah N. Boers, Karin R. Jongsma, Federica Lucivero, Jiska Aardoom, Frederike L. Büchner, Martine de Vries, Persijn Honkoop, Elisa J. F. Houwink, Marise J. Kasteleyn, Eline Meijer, Hilary Pinnock, Martina Teichert, Paul van der Boog, Sanne van Luenen, Rianne M. J. J. van der Kleij, Niels H. Chavannes

**Affiliations:** a Julius Centre for Health Sciences and Primary Care, Department of Medical Humanities, University Medical Centre Utrecht, Utrecht, the Netherlands;; b Ethox and Wellcome Centre for Ethics and Humanities, Nuffield Department of Population Health, University of Oxford, Oxford, UK;; c Department of Public Health and Primary Care, Leiden University Medical Centre, Leiden, the Netherlands;; d National eHealth Living Lab (NELL), Leiden, the Netherlands;; e Department of Medical Ethics and Health Law, Leiden University Medical Centre, Leiden, the Netherlands;; f Biomedical Data Sciences, Leiden University Medical Centre, Leiden, the Netherlands;; g Usher Institute of Population Health Sciences and Informatics, University of Edinburgh, Edinburgh, UK;; h Department of Clinical Pharmacy & Toxicology, Leiden University Medical Centre, Leiden, the Netherlands;; i Department of Nephrology, Leiden University Medical Centre, Leiden, the Netherlands

**Keywords:** eHealth, digital health, primary care, ethics, doctor–patient relationship

## Abstract

**Background:** eHealth promises to increase self-management and personalised medicine and improve cost-effectiveness in primary care. Paired with these promises are ethical implications, as eHealth will affect patients’ and primary care professionals’ (PCPs) experiences, values, norms, and relationships.

**Objectives:** We argue what ethical implications related to the impact of eHealth on four vital aspects of primary care could (and should) be anticipated.

**Discussion:** (1) EHealth influences dealing with predictive and diagnostic uncertainty. Machine-learning based clinical decision support systems offer (seemingly) objective, quantified, and personalised outcomes. However, they also introduce new loci of uncertainty and subjectivity. The decision-making process becomes opaque, and algorithms can be invalid, biased, or even discriminatory. This has implications for professional responsibilities and judgments, justice, autonomy, and trust. (2) EHealth affects the roles and responsibilities of patients because it can stimulate self-management and autonomy. However, autonomy can also be compromised, e.g. in cases of persuasive technologies and eHealth can increase existing health disparities. (3) The delegation of tasks to a network of technologies and stakeholders requires attention for responsibility gaps and new responsibilities. (4) The triangulate relationship: patient–eHealth–PCP requires a reconsideration of the role of human interaction and ‘humanness’ in primary care as well as of shaping Shared Decision Making.

**Conclusion:** Our analysis is an essential first step towards setting up a dedicated ethics research agenda that should be examined in parallel to the development and implementation of eHealth. The ultimate goal is to inspire the development of practice-specific ethical recommendations.

KEY MESSAGES

- The impact of eHealth on primary care is paired with ethical implications including questions of autonomy and professional responsibilities
- Practice-specific ethical guidance for the use of eHealth in primary care should be developed
- Primary care professionals should be aware of the ethical implications when using eHealth approaches

## Introduction

Information and Communication Technologies (ICT) are increasingly permeating every aspect of our lives. Algorithms determine the advertisements to which we are exposed, our phones switch our house lights on and off, and wearable sensors track our sleeping patterns. Not surprisingly, ICT also permeate health care in the form of eHealth. EHealth encompasses a broad category of ‘*health services and information delivered or enhanced through the internet and related technologies’* that are employed to support or improve health care [1]. Examples include health apps, wearables and tracking devices, monitoring at a distance and digital communication interfaces. EHealth is expected to change primary care dramatically. Not only do applications developed by the health care sector formally enter the consultation room (Box 1) [2–5], also commercially available eHealth applications and wearable devices used by patients impact their interactions with healthcare professionals [6,7].

How should we value these developments? Generally, eHealth promises to increase self-management and personalised medicine, empower patients, and improve cost-effectiveness [4,8,9]. eHealth supporters stress that the technology will contribute to an ideal healthcare system in which patients are empowered and the centre of their personal continuum of care [10]. However, it can also be argued that rather than unleashing a revolution, eHealth is supportive of core values and activities of primary care professionals (PCPs), who have always focussed on encouraging self-management and delivering person-centred and continuous care [11]. eHealth critics stress the downsides of eHealth, such as the potential loss of human interaction, the lack of validation from algorithms used, commercial interests, and a lack of confidentiality in commercially available applications [12–14]. Furthermore, critical voices mention that most eHealth solutions today still lack a solid evidence base; that uptake by PCPs is still limited; that eHealth raises privacy and informed consent concerns [4,12]. The latter have drawn considerable attention in both academic and popular media, particularly in light of the recent General Data Protection Regulation (GDPR) [15].

The ethical implications of eHealth, however, extend well beyond privacy and informed consent issues [6,9,16–18]. As new health technologies affect users’ experiences, values, norms, and relationships [19,20], the increasing use of digital technologies in primary care can be expected to profoundly affect professionals and patients in- and outside of the consultation room. These changes can affect day-to-day primary care practice and raise ethical questions that require attention. Therefore, in this paper, we discuss what ethical implications could (and should) be anticipated in four essential aspects of primary care: (1) dealing with predictive and diagnostic uncertainty; (2) the roles and responsibilities of (future) patients; (3) the roles and responsibilities of PCPs; (4) the patient – PCP relationship. We construct our arguments by drawing upon the academic literature regarding eHealth, ethics, digitalisation, and primary care as well as upon our diverse experiences and expertise regarding the development, ethical evaluation, implementation, and use of eHealth in primary care. Subsequently, we suggest ways forward by listing a set of empirical, conceptual, and normative ethics research questions that need to be addressed. In addition, we propose a method for the ethical accompaniment of the design and implementation of novel eHealth tools. Finally, we emphasise the importance of these ethical dimensions for PCPs when using eHealth approaches in their day-to-day practice.

## Ethical implications of the clinical use of eHealth in primary care

### Dealing with predictive and diagnostic uncertainty

One of the core tasks of PCPs is to diagnose diseases at an early stage to provide timely treatment and ideally to predict and prevent disease. When executing this task PCPs have to deal with substantial uncertainty. In the early phases of disease, symptoms are undifferentiated, and PCPs have limited predictive and diagnostic possibilities, some of which have limited accuracy [21].

Box 1 Potential applications of eHealth in primary care

- eHealth enables patients to monitor their (un)healthy behaviour and to spur lifestyle interventions and behavioural change [7,8,16]. A plethora of apps, wearables, and online platforms aim to increase self-management [32]. Examples include monitoring of adherence to medication and remote monitoring of patients with complex medical and social problems at home instead of in the hospital [33].
- eHealth approaches can facilitate and improve the interaction between patients and healthcare providers and among healthcare professionals [7]. Online consultation via patient portals and digital communication interfaces, such as teleconferencing and online patient access to medical files, are increasingly adopted.
- Data are expected to inform medical decision-making [7]. Patients increasingly become generators of data via apps, wearables, and smartphones. These data, if combined with other types of information (e.g. information derived from genomics, environmental and socio-cultural data) and machine learning approaches, can personalise medical decision-making.

EHealth can alter how PCPs deal with such uncertainties. Machine learning approaches can use large amounts of data to predict or diagnose disease more accurately through a quantified and personalised calculation. When integrated with clinical decision support systems (CDSSs), these machine learning approaches function as decision aids [22].

Whereas eHealth may solve some aspects of uncertainty, at the same time novel loci of uncertainty are introduced, because the decision-making process becomes opaque (this is sometimes referred to as a ‘black-box’) [22–24]. The quality of the data may be unclear, and the algorithms and weighting of different parameters and trade-offs may not be transparent. This is particularly so because in machine learning the system changes and develops over time depending on the data to which it is exposed [23].

The described opacity has several ethical implications. First, it makes it difficult for PCPs to judge the validity of the outcome, among others because sensitivity and specificity of a test cannot be calculated, this while the validity of algorithms may well be compromised [23]. This not only leads to the epistemological question of what evidence-based practice in primary care is, but it can also conflict with some of the principle moral duties of PCPs’, namely to do no harm and to do good to their patients. If you cannot judge whether algorithms make valid and fair decisions, how can you make sure that you use the decision aid responsibly? [23] Moreover, it can be unclear whether the outcome applies to the specific patient in the consultation room, which may hamper person-centred care. Certain groups may have been excluded from the data analysis as bias and discrimination can be inherent to machine learning approaches [22]. This potentially threatens the PCP’s duty to secure equal accessibility for their patients. Lastly, if the algorithms are embedded in CDSSs that generate recommendations regarding diagnostic follow-up or treatment, the system provides a seemingly objective choice that is in fact value-laden [24]. In developing rule-based algorithms implicit or explicit choices are made regarding e.g. acceptability of risk or treatment preferences. These choices require a trade-off between different values. If ‘safety first’ is the mandate, cut-off points for referral may differ from a situation in which cost-effectiveness is the guiding value. This raises the question of what should be guiding values and who should decide the norm [22,24,25]. Also, PCPs and patients need to know how algorithms determine their recommendations in order to allow for autonomous decision-making [24]. However, this is precisely the difficulty with opaque machine learning systems (Table 1).

**Table 1.** The ethical implications of the clinical use of eHealth in primary care.

	Changes	Ethical implications
1. Dealing with predictive and diagnostic uncertainty	Machine learning based CDSSs Personalised and quantified prediction, diagnosis and treatment ranking Opaque decision-making processes	Valid and good clinical judgment Duty to do good and to do no harm Bias and discrimination Inclusivity and equality of care Value-laden choices
2. The roles and responsibilities of patients	Self-management Personalised lifestyle interventions	Autonomy (Moral) responsibilities Equality and inclusivity
3. The roles and responsibilities of PCPs	Delegation of tasks to a network of technologies and stakeholders (Persuasive) lifestyle interventions	Responsibility gaps Novel responsibilities New balance between the duty to do good and fostering patient autonomy
4. The patient-PCP relationship	Triangulate relationship patient-PCP-eHealth (Partial) replacement direct contact by eHealth	‘Humanness’ and quality of care Shared decision making Trust and confidentiality

### Roles and responsibilities of patients

EHealth approaches influence the autonomy, roles, and responsibilities of patients, because of how they can promote self-management and lifestyle interventions [8,9,18].

The use of eHealth to enhance self-management fits well within the scope of primary care, where patient empowerment and the stimulation of patient autonomy are part of the core values [11]. Diagnosing or monitoring disease without the participation of PCPs can make patients more involved in their own care [23]. Also, patients can become more familiar with their own body and disease symptoms, and become increasingly able to respond appropriately to changing symptoms [2,16,17]. Lifestyle trackers can help patients to meet their own goals [12,26]. On the flipside, patient autonomy can be compromised. For lifestyle applications to enhance autonomy, patients should be able to set their own goals, it should be transparent how advice is generated, and patients should be able to choose whether or not to respond to advice [26]. Some of the currently available lifestyle applications lack these characteristics. Examples include applications that automatically adjust the daily goals based on a real-time feedback loop of patient’s data and online behaviour [12].

Furthermore, if PCPs attribute new responsibilities to patients through employing eHealth, they should be aware that eHealth-mediated self-management could also be harmful. It can be (too) burdensome, the quality of measurements can decrease, and it can cause feelings of isolation unless eHealth offers the possibility to share worries and seek immediate contact [18]. Also, in some cases the (moral) responsibility of patients for the quality of measurements, the adherence to medication or lifestyle interventions, or for choosing (not) to consult a physician or go to work can be unjustly heavy, particularly if factors that are beyond their control are insufficiently taken into account.

Besides, eHealth approaches raise questions of accessibility, equality, and inclusivity of care [27]. The primary care population is highly heterogeneous and includes patients from diverse cultural backgrounds, with different socioeconomic status, health literacy, educational level, and age groups. EHealth applications may not be equally accessible to all of these groups, either because they are unsuitable or unaffordable. This may increase existing health disparities [27]. Together with potential bias and discrimination in algorithms these aspects may raise issues of representativity and exclusion of certain groups from appropriate healthcare provision.

### Roles and responsibilities of PCPs

If PCPs use eHealth, they delegate tasks to a network of technologies (e.g. to CDSSs and remote monitoring technologies) and people (e.g. designers, data scientists, patients, other caregivers). This delegation of tasks urges a reconsideration of professional responsibilities [22,28].

If PCPs delegate tasks to eHealth applications such as remote monitoring technologies, new responsibilities arise. For example, in applications that have a triage function, who is responsible for monitoring the monitors? Also, PCPs should be aware of potential responsibility gaps [29]. Who is responsible or accountable if an alert generated by a remote monitoring technology, such as the home monitoring of cardiac rhythm, is missed: the patient, the PCP, the manufacturer, the digital infrastructure, or the data scientist? These are pressing questions, among others, because current digital systems frequently have insufficient interoperability.

Additionally, the ways PCPs fulfil their duty to foster the autonomy of their patients should be reconsidered. PCPs should have extra attention for those patients that have low health or digital literacy. Moreover, an adequate balance should be struck between using persuasive lifestyle applications in the best interest of the patient and fostering patient autonomy. Although PCPs have a duty to do good, we should be weary of a return to paternalistic medicine, for instance through strongly persuasive lifestyle applications (see previous paragraph) or through value-laden decision aids (see next paragraph) [24,29].

### Patient–PCP relationship

When using digital technologies in primary care, a triangular relationship of patients –eHealth – PCPs comes into being. This means that the direct interaction between patients and PCPs can be influenced by eHealth, e.g. if face-to-face contact is replaced by an eHealth medium or if a CDSS is used to support decision-making [3,22]. Simultaneously, PCPs may be bypassed if eHealth is employed by patients for self-management. These changes have ethical implications, for example regarding the ‘humanness’ of primary care and the role of trust and confidentiality.

Replacement of face-to-face contact by eHealth implies that the role, importance, and meaning of human interaction in primary care must be reconsidered [11,23]. Attention for the human elements of care and a holistic attitude are considered to be core values of primary care [11]. Direct human interaction between PCPs and patients has a vital role in shaping these attitudes. Furthermore, the contact between the doctor and the patient can influence how a patient responds to illness and treatment [30]. Also, direct interaction is necessary for some aspects of adequate diagnosis and triage, such as an overall sensory impression of the patient (e.g. smell) and physical examination [18]. The (partial) replacement of direct human interaction by eHealth may undermine these aspects of primary care. Alternatively, eHealth may lead to a reinterpretation of (or a complementary approach to) human interaction. Patients could, for example, feel more at ease discussing sensitive topics, such as psychological or sexual complaints, via a digital interface [18]. Notwithstanding, attention should be paid to upholding the ‘humanness’ of primary care. To this end, it should be carefully considered what types of care can be delegated to eHealth approaches without losing quality or compromising safety (e.g. in case of triage) and how eHealth can be blended with face-to-face care [4].

Furthermore, eHealth may support, strengthen, or compromise Shared Decision-Making (SDM) [24]. SDM is increasingly considered of crucial importance in primary care [11]. EHealth can improve SDM processes, because it can stimulate self-management and –monitoring and it makes medical knowledge more widely available. A combination of increased self- and medical knowledge can enable patients to identify and articulate their goals, values, and preferences, which spurs the SDM process. Furthermore, if algorithms generate personalised risk scores or treatment options, these outcomes are quantified and made explicit, which could be the starting point of an SDM discussion. Alternatively, eHealth may compromise SDM. CDSSs can generate seemingly objective and personalised rankings, for instance, regarding the preferred treatment modality [24]. However, these rankings are based on general decision principles that do not necessarily align with the patient’s preferences. Although patients can decide to deviate from these recommendations, the apparent objectivity of algorithms may make this problematic. Also, in SDM ideally the values of patients serve as a starting point for the process, rather than an aftermath [24].

Lastly, eHealth can affect the notions of trust and confidentiality within the patient-physician relationship. Trust in clinical judgment can be either enhanced because CDSSs are (seemingly) objective, or decreased because of the opaque decision-making process [23]. Additionally, most eHealth applications are developed by commercial companies whose business model is frequently based on the collection, use, and sale of data [6]. This reality changes the nature of confidentiality in primary care. It should be discussed to what extent PCPs have a duty to judge the trustworthiness of commercially available apps and to guide their patients in responsible and safe use of these applications.

## Towards morally responsible innovation and implementation of eHealth

The ethical implications we have discussed form an essential first step towards developing practice-specific ethical guidance for the implementation and use of eHealth in primary care.

We suggest that to proceed forward, a dedicated ethics research agenda should be set up addressing empirical, conceptual, and normative questions (Table 2). Empirical questions encourage an investigation of how eHealth affects primary care (e.g. how specific tools enable patients to self-manage their conditions and how this influences their perception of autonomy). Conceptual questions focus on the reinterpretation of moral principles and norms in medical ethics in the digital era. Lastly, normative questions concern the desirability and moral acceptability of specific e-health practices. Examples of questions in Table 2 serve as a stepping-stone to set up a dedicated ethics research agenda for different types of eHealth.

**Table 2.** Examples of potential empirical, conceptual, and normative questions.

	Examples of potential research questions
Empirical: what impact do eHealth technologies have on disease perception, roles and responsibilities, relationships, values, experiences etc.?	1. How does personalised quantification of cancer risk via clinical decision support systems (CDSS) influence the shared decision-making process between patients and primary care providers? 2. How do apps that aim to improve self-management of chronic diseases influence the perceived self-management and autonomy of patients? 3. How do algorithms that partially black-box decisions affect the clinical judgment of primary care providers?
Conceptual: how should we *understand* the shifts in disease perception, roles and responsibilities, relationships, values, and experiences etc.?	1. How should we conceptualise the way cancer prediction models frame risk? How does this relate to existing concepts of risk? 2. How should we understand patient autonomy in the digital era? 3. How should we conceptualise responsibility for clinical judgment when using black-box algorithms?
Normative: how should we *deal with* the shifts in disease perception, roles and responsibilities, relationships, values, and experiences etc.?	1. What are appropriate cut-off points for cancer risks in CDSS to decide upon referral for further cancer diagnostics? Who should decide what the appropriate cut-off points are? 2. To what extent is persuasive technology in apps that aim to improve self-management in chronic diseases justifiable? 3. What level of transparency for algorithms in CDSS is required?

Moreover, we contend that, because eHealth technologies fundamentally change primary care practice and values, there should be attention for these ethical questions already during the development, design, and implementation process. This goal can be achieved through ‘parallel ethics research’ [19,31]. In this approach ethicists, together with other stakeholders (e.g. PCPs, patients, biotech companies, data scientists), identify and evaluate the ethical challenges associated with an eHealth technology parallel to its development and implementation [19,31]. The output of this interdisciplinary ethical analysis can directly influence the design of the eHealth intervention. If ethical implications specific to the eHealth intervention are anticipated (e.g. a potential compromise of patient autonomy) and desirable use is defined (e.g. stimulating patient autonomy and improving lifestyle), the eHealth intervention can be designed such that the ethical hurdles are avoided and desirable use is propagated. Additionally, conditions can be formulated to ensure that the technology is embedded responsibly in the healthcare system (e.g. conditions for shaping blended care and improving SDM).

Additionally, we advise that formal spaces be created for PCPs to raise awareness of the impacts of eHealth on their own and their patients’ norms and values when using eHealth approaches in their day-to-day practice. To this end, we recommend the setup of specific training and learning opportunities. In a follow-up paper in this eHealth series, we will further elaborate on the newly required professional skills and educational needs.

## Concluding remarks

In this paper, we have made the first step towards morally responsible use of eHealth in primary care by providing an exploration of its ethical implications in primary care practice. EHealth may not necessarily revolutionise primary care, but it does bring about necessary reconfigurations of the practice. We have identified four areas of change that need attention, specifying the ethical implications that result from each one of them. Our analysis has shown that eHealth approaches call for a reconsideration of some of the core moral values inherent to primary care. We have also delineated the boundaries for a much-needed dedicated ethics research agenda, providing examples of empirical, conceptual, and normative research questions specific to the use of eHealth in primary care. This is an essential first step towards the formulation of practice-based and practice-specific ethical recommendations for the clinical implementation and use of eHealth in primary care.
